# Zinc and Copper with New Triazine Hydrazone Ligand: Two Novel Organic Complexes Enhanced Expression of Peptide Growth Factors and Cytokine Genes in Weaned V-Line Rabbit

**DOI:** 10.3390/ani9121134

**Published:** 2019-12-12

**Authors:** Abdelmotaleb A. Elokil, Tharwat A. Imbabi, Hany I. Mohamed, Khaled F. M. Abouelezz, Omar Ahmed-Farid, Girmay Shishay, Islam I. Sabike, Huazhen Liu

**Affiliations:** 1 Department of Basic Veterinary Medicine, College of Animal Science and Veterinary Medicine, Huazhong Agricultural University, Wuhan 430070, China; shishay@webmail.hzau.edu.cn; 2 Animal Production Department, Faculty of Agriculture, Benha University, Moshtohor 13736, Egypt; tharwat.mohamed@fagr.bu.edu.eg; 3 Department of Chemistry, College of Science, Huazhong Agricultural University, Wuhan 430070, China; hany.ibrahim@mail.hzau.edu.cn; 4 Chemistry Department, Faculty of Science, Benha University, Benha 13518, Egypt; 5 Institute of Animal Science, Guangdong Academy of Agricultural Sciences, Dafeng Street 1, Wushan, Tianhe District, Guangzhou 510640, China; abollez@aun.edu.eg; 6 Department of Poultry Production, Faculty of Agriculture, Assiut University, Assiut 71526, Egypt; 7 Department of Physiology, National Organization for Drug Control and Research, Giza 12553, Egypt; ebntaimya@yahoo.com; 8 Department of Food Hygiene, Faculty of Veterinary Medicine, Benha University, Moshtohor 13736, Egypt; Islam.sabek@fvtm.bu.edu.eg

**Keywords:** zinc and copper supplementation, organic feed supplements, triazine hydrazone complex, peptide growth factor expression, rabbit growth rate

## Abstract

**Simple Summary:**

Zinc and Copper are two important trace minerals, which are involved in numerous vital biological activities in animal’s body, such as enzyme activation and enhancement of immune function, growth, reproduction, DNA synthesis, cell division, and neurotransmitters production. Recently, the application of trace mineral organic complexes in animal feed received much more attention than the inorganic sources. The organic sources can contribute significantly to improving animals’ health and reproduction, as the minerals are more biologically available and absorbable than they are when coming from the inorganic sources. In this study, three injectable varieties of Zn and Cu supplementation, sulfate, inorganic (loaded with montmorillonite), and novel organic (ligand with triazine hydrazone), were tested with weaned rabbits. The results revealed that these three mineral types vary at the most biological responses, and only one category of our novel organic complexes provided consistent animal performance improvement, including weight gain, serum antioxidant, meat quality, intestine morphometry, and the expression of peptide growth factors and cytokine genes. To our knowledge, this is the first work on the Zn and Cu with triazine hydrazone ligand as two organic complexes in rabbits.

**Abstract:**

Two novel transitional organic Zn/Cu complexes based on a new biocompatible bidentate triazine–hydrazone ligand (Thz) was designed, synthesized, and evaluated in this study. This study evaluated the effects of injecting 60 mg of Zn and 40 mg of Cu in three different forms, twice per week, for eight weeks on growth performance, expression of growth factors and cytokine genes, carcass yield, blood biochemicals, and intestinal morphology in weaned rabbits. The tested complexes were sulfate (Cu/ZnSO_4_), montmorillonite (Cu/Zn-Mnt), and triazine hydrazone (Cu/Zn-Thz). A total of 60 V-line weaned rabbits at four weeks of age were assigned to four treatments (*n* = 15), which were intramuscularly injected with 0.5 mL of either (1) saline (control) or saline containing (2) Cu/ZnSO_4_, (3) Cu/Zn-Mnt, or (4) Cu/Zn-Thz. Compared to the controls, the rabbits injected with Cu/Zn-Thz showed a higher (*p* < 0.01) growth rate, carcass yield (*p* < 0.05), and liver expression of insulin like growth factor-1 (*IGF-1*), growth hormone receptor (*GHR*), fibroblast growth factor-1 (*FGF1*), and transforming growth factor beta-1 (*TGFB1*) (*p* < 0.05), as well as better jejunum morphometric variables (*p* < 0.05). On the other hand, mRNA of *FGF1*, *TGF1*, *TCIRG1*, and adenosine deaminase (*ADA*) were higher expressed (*p* < 0.05) in the spleen tissues of Cu/Zn-Mnt group. Collectively, the results indicated that our novel synthesized organic complexes of Zn/Cu-Thz proved to be a suitable feed supplement, as it increased rabbit productive performance through enhancing expression of peptide growth factors and cytokine genes.

## 1. Introduction 

Trace elements, especially copper (Cu) and zinc (Zn), have vital structural or catalytic roles in many metal-binding proteins and metalloenzymes, which are important for immune system function, nutrient metabolism [1,2,3], and health in rabbits; in addition, they had proven significant antimicrobial effect [4,5,6]. Pharmacological amounts of Zn and Cu, either incorporated into the diet or injected into the body, enhanced the growth performance of rabbits, particularly after/during the weaning stage [7]. The various sources of supplemental trace minerals and particularly Zn and Cu, such as oxide, nanoparticles, carrier loading, and ligand with complexes, showed different effects on animals’ growth rate; some sources have a poor mineral bioavailability, leading to irritation in the intestinal mucosa or increase in the excretion of trace minerals to the intestinal environment [8]. In poultry, the organic dietary sources of Zn and Cu show a higher mineral bioavailability when compared with that of the inorganic resources in poultry [1,2,3] and pigs [9].

Montmorillonite (Mnt), being a natural silicate clay, possess a variety of excellent properties that make it a good drug-delivery carrier. It, therefore, could be used as an effective carrier for Cu and Zn, increasing their biological activity in animals, by increasing their bioavailability [10,11]. It was reported that loading metal ions onto montmorillonite (Mnt) displayed obvious synergistic antimicrobial effects in vitro [12,13]. Metal-loaded Mnt, in addition, has a high availability of metals, with great safety, biological activity, and long-term effectiveness [14]. On the other hand, organic ligands, especially hydrazones, can effectively trap metals via coordination, and some triazine hydrazones have been reported to exhibit a selective recognition to a specific G-quadruplex DNA structure [15]. Based on their good biocompatibility that make their absorption easier, their complexes with metals have a double function. In some cases, they have desirable medicinal effects [16], aside from their main role as element supplement sources. To the best of our knowledge, no previous studies investigated the importance of such complexes as nutritional supplements in poultry. It is, therefore, anticipated that this type of compound may serve as an acceptable source of metals.

The objective of this study was to evaluate the effects of injecting Cu and Zn loaded on three forms, Zn/CuSO_4_, Zn/Cu-Mnt (Mnt as an inorganic carrier), and Zn/Cu in triazine hydrazone complex (Thz as an organic complex), on the expression of *IGF-1*, *GHR*, *FGF1*, and *TGFB1*, as well as cytokine genes (*TCIRG1*, *IL10*, and *ADA*), intending to improve the growth performance of growing rabbits. 

## 2. Materials and Methods

### 2.1. Ethics Approval and Consent to Participate

For the experimental field on animal, all protocols for all animal experiments were approved by the Scientific Ethic Committee of Animal Production Department, Faculty of Agriculture, Benha University, Egypt. The laboratory experiments and protocols were handled in accordance with the guidelines described by Huazhong Agricultural University, Hubei Province, P.R. China.

### 2.2. Preparation of Modified Mnt (Zn-Mnt and Cu-Mnt)

The modified Mnt was prepared via ion-exchange reactions, as described previously by Jiao et. al. [10]. Briefly, 10.0 g of Mnt was added portion-wise to a stirred 0.2 M of NaCl solution (100 mL). Agitation was continued for 8 h, at 600 rpm, and the Na-Mnt was separated by centrifugation, washed many times with deionized water, oven-dried, and then ground to powder. Na-Mnt (2.0 g) was added to 50 mL of a 0.2 M solution of zinc sulphate or copper sulphate. To increase the rate of the cation exchange, each dispersion was stirred at 60 °C for 8 h. Centrifugation was also used to separate the sediments that were washed with deionized water. Overnight drying at 80 °C and grinding produced the powder. Zn-Mnt; off-white, FT-IR (KBr, cm^−1^) υ 3624, 3457, 1638, 1087, 1035, 914, 843, 794, 624, 519, and 466. Cu-Mnt; cyan, FT-IR (KBr, cm^−1^) υ 3624, 3447, 1637, 1092, 1034, 914, 843, 795, 623, 522, and 471. 

### 2.3. Synthesis of the Thz Ligand

**Figure 1:**
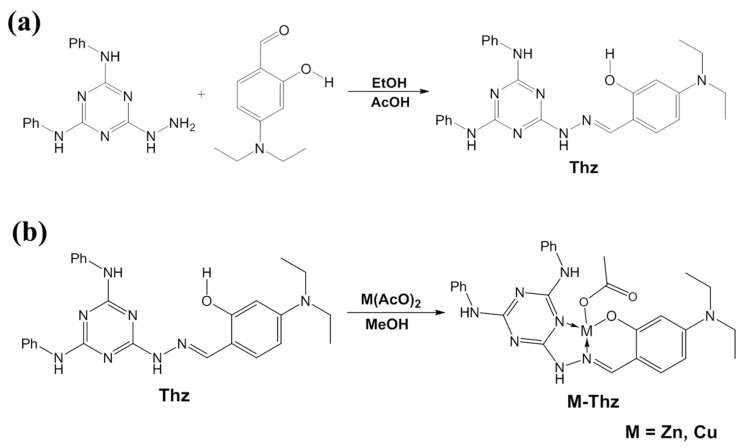
(**a**) Scheme 1: synthetic route of triazine hydrazone (Thz). (**b**) Scheme 2: synthesis of Thz complexes with zinc and copper.

**Figure 2:**
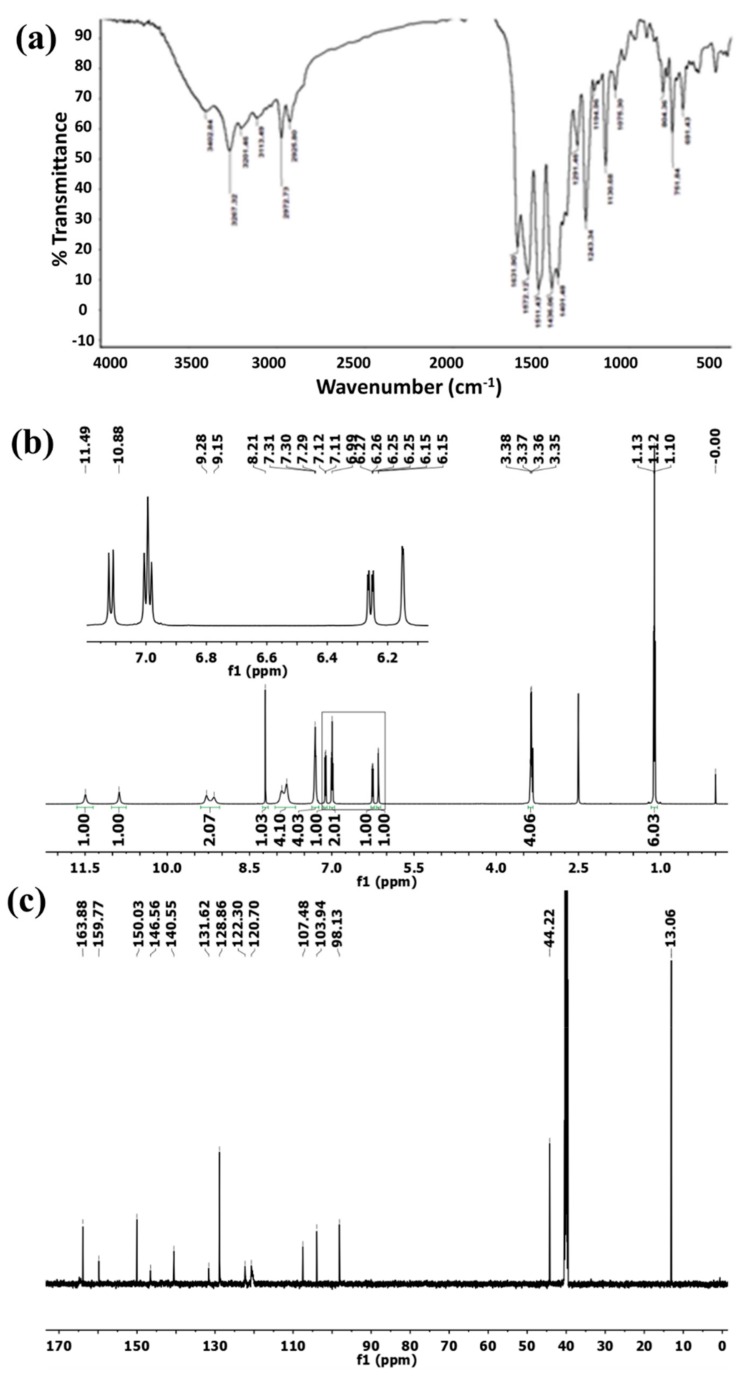
Spectroscopic characterization of the ligand; Thz. (**a**) FT-IR, (**b**) ^1^H NMR, and (**c**) ^13^C NMR spectra.

This compound was synthesized according to Scheme 1 (Figure 1a). As is typical, a total of 586 mg (2 mmol) of 6-hydrazinyl-N2, N4-diphenyl-1,3,5-triazine-2,4-diamine [17] was suspended in 25 mL of absolute ethanol, and 386 mg (2 mmol) of 4-(diethylamino) salicylaldehyde and glacial acetic acid (3 drops) were added. The mixture was then refluxed at 80 °C for 3 h. The precipitated product was filtered, washed with petroleum ether, and air-dried to afford ***Thz*** as an off-white solid yielding 640 mg (68%). FT-IR (KBr, cm^−1^) υ 3403, 3267, 3201, 3114, 2973, 2926, 1632, 1572, 1511, 1436, 1402, 1243, and 1131. ^1^H NMR (600 MHz, DMSO-d_6_) δ 11.49 (s, 1H), 10.88 (s, 1H), 9.28 (s, 1H), 9.15 (s, 1H), 8.21 (s, 1H), 7.91 (s, 2H), 7.82 (s, 2H), 7.30 (t, *J* = 6.6 Hz, 4H), 7.12 (d, *J* = 8.7 Hz, 1H), 6.99 (d, *J* = 7.3 Hz, 2H), 6.26 (dd, *J* = 8.7, 2.4 Hz, 1H), 6.15 (d, *J* = 2.0 Hz, 1H), 3.36 (q, *J* = 7.0 Hz, 4H), and 1.12 (t, *J* = 7.0 Hz, 6H). ^13^C NMR (150 MHz, DMSO-d_6_) δ 163.88, 159.77, 150.03, 146.56, 140.55, 131.62, 128.86, 122.30, 120.70, 107.48, 103.94, 98.13, 44.22, and 13.06 (Figure 2a–c).

### 2.4. Synthesis of M–Thz Complexes

The two complexes were synthesized, as depicted in Scheme 2 (Figure 1b). A mixture of *Thz* (300 mg, 0.64 mmol) and metal acetates, namely zinc acetate dihydrate (145 mg, 0.66 mmol) or copper acetate (120 mg, 0.66 mmol) in 25 mL of methanol, was heated, with stirring at 70 °C. A clear solution (yellow in the case of zinc, and green in the case of copper) was observed after 20 min. After refluxing for 3–4 h, a new precipitate was formed that was collected by filtration and dried. *Zn-Thz*, yellow crystals, FT-IR (KBr, cm^−1^) υ 3264, 2965, 2922, 1662, 1595, 1512, 1440, 1348, 1240, and 1133. *Cu-Thz*; dark-green crystals, FT-IR (KBr, cm^−1^) υ 3270, 2968, 2926, 1666, 1597, 1516, 1443, 1351, 1242, and 1135.

### 2.5. Animals and Experimental Design

A total of 60 V-line weaned rabbits aged four weeks, having a similar body weight average (400 ± 20 g), were divided into four groups (*n* = 15) and housed in twenty replicate cages (45 × 55 × 30 cm), with each holding three animals; five cages were randomly assigned to one of four treatments. The first group of animals was intramuscularly injected with 0.5 mL of saline solution (0.9% NaCl); the other three groups were injected with 0.5 mL of saline containing 60 mg of zinc and 40 mg of Cu, either in the form of Cu/ZnSO_4_, Zn/Cu-Mnt, or Zn/Cu-Thz. All animals were subcutaneously injected, twice weekly (each Saturday and Tuesday), during the experimental period (8 weeks), between 4 and 12 weeks of age. All animals were fed the same standard iso-caloric/iso-nitrogenic diet during the experimental period. The basal diet composition and calculated analysis followed the nutrient requirements of rabbits from the National Research Council (NRC) [18], as shown in Table 1. 

### 2.6. Growth Performance and Carcass Evaluation

The initial and final body weights (BW) were weekly recorded on an individual basis, using a digital balance, and the average daily weight gain (ADG) was calculated as the difference between final and initial BWs divided by the number of days of the experimental period. At the end of the experimental period, a total of five animals (*n* = 5, the average weight of their group) were selected for all further experiments; the animals were slaughtered to evaluate the carcass traits and weight of internal organs. The weights of each carcass, abdominal fat, hind legs, saddle, thoracical neck, liver, kidney, spleen, and lung were recorded and expressed vis-à-vis the final body weight. 

### 2.7. Physicochemical Analysis of Musculus Longissimus Dorsi (MLD)

Physicochemical analysis was conducted on *Musculus longissimus dorsi* (MLD) of the right and left sides of rabbit (*n* = 5) carcasses. The pH grade (pHU) was measured directly on MLD, after 24 h, using a digital pH-meter (Thermo Orion 710 A+, Cambridgeshire, UK). Water holding capacity (WHC) was determined at 24 h after slaughter, by the low-speed centrifugation method at 10,000 rpm and 5 °C, for 20 min [19]. The drip loss (at 24 and 48 h) and cooking loss were evaluated according to the method described by Honikel [20]. Warner–Bratzler shear force (WBSF) was measured (3343 universal test system mono column, Instron, USA) according to the American Meat Science Association (AMSA) [21]. A chroma meter CR-410 (Konica Minolta Sensing INC., Osaka, Japan) was used to assess lightness (L*), redness (a*), and yellowness (b*) of MLD. These color scores, therefore, were used for calculation of color chroma as (C = (a*^^2^ + b*^^2^) ^^0.5^) and color saturation as (Hue angle, h˚ = arctg b*/a*). Total aerobic plate count (APC) and total staphylococcus count of meat were enumerated after incubation at 37 °C, for 48 h, on an aerobic plate count agar and Baird–Parker agar, respectively [22].

### 2.8. Antioxidant Variables Assay

At the end of the experimental period, blood samples for five animals of each group (*n* = 5, the average weight of their group) were collected from the marginal air vein of the ears into non-heparinized tubes (10.0 mL). Thereafter, fresh serum samples were prepared by centrifugation at 3000 rpm for 12 min in the laboratory. The concentration of malondialdehyde (MDA; biological marker of cell membrane degradation), adenosine monophosphate (AMP; biological marker of cell energy degradation) and endogenous nonenzymatic antioxidant of reduced glutathione (GSH), and oxidized glutathione (GSSG) were detected in serum samples (*n* = 5) by HPLC method (Agilent HP 1200 series HPLC apparatus, Midland, ON, Canada), according to Jayatilleke and Shaw [23] and Karatepe [24]. All standards (MDA: 100683-54-3; AMP: Number: 4578-31-8; GSH: 200-725-4; GSSG: 103239-24-3) were obtained from Sigma-Aldrich Chemie GmbH Export Co. Ltd., Taufkirchen, Germany. Additionally, the serum activates of catalase (CAT) and superoxide dismutase (SOD) were measured by spectrophotometric method, at 420 nm, for 1 min, on a UV-Vis Shimadzu spectrophotometer (2450) [25].

The concentration of 8-hydroxy-2′–deoxyguanosine (8-OHdG) in brain tissue (*n* = 5) was measured to assess oxidative stress and carcinogenesis. Briefly, genomic DNA was extracted from brain tissue by using a kit (QIAGEN, Hilden, Germany), according to the manufacturer recommendations. The hydrolyzed mixture was centrifuged, and the supernatant was injected into the HPLC. The separation of 8-OHDG was performed with an LC/Agilent 1200 series HPLC apparatus (USA), using a UV detector. For chromatographic separation, we used C18 reverse-phase columns in series (Supelco, 5 pm, I.D. 0.46 × 25 cm); the eluting solution was H_2_O/CH_3_OH (85:15 v/v), with 50 mM of KH_3_PO_4_, pH 5.5, at a flow rate of 0.68 mL/min. The UV detector was set at 245 nm. The resulting chromatogram was used to identify the concentration of the sample as compared to that of the standard purchased from Sigma-Aldrich.

### 2.9. Muscle Amino Acid Profile Assay

Each MLD muscle sample (*n* = 5) was weighed and homogenized in 75% aqueous HPLC grade methanol (10% w/v). The homogenate was spun at 4000 rpm/10 min, and the supernatant was dried with a vacuum (70 Millipore), at room temperature, and used for amino acid (AA) assay by HPLC, using the precolumn PITC derivation technique [26].

### 2.10. Quantitative Histomorphometric Analysis of Jejunum Segments

Segments of the mid-jejunum (3 cm) were collected from five animals per group, fixed with formalin for 48 h, and paraffin-embedded. Two sections (100 μm) from each sample were obtained, stained with hematoxylin for 1 min, and counterstained with eosin for 10 s, to assess the maximum villus length (measured from above the crypt to the tip of the villus), crypt depth, and submucosa/muscularis/serosa thickness. All targeted variables were measured with a camera (OLYMPUS; TH4-200; Tokyo, Japan) coupled with computer-assisted digital-image pro plus (IPP) analysis software (Image-Pro Plus 4.5, Media Cybernetics, Silver Spring, MD, USA).

### 2.11. Tissues Collection, RNA Isolation and cDNA Synthesis

A total of five samples from both liver and spleen tissue were immediately collected after slaughtering each group. The samples were suddenly frozen in liquid nitrogen and stored at −80 °C until the RNA isolation. Total RNA was independently extracted from liver and spleen samples (*n* = 5), using the TRIzol reagent (Invitrogen, Carlsbad, CA, USA), according to the manufacturer instructions, and then RNA was purified with DNase I (TaKaRa, Shiga, Japan) and an RNA clean kit (TIANGEN, China). The quantity and quality of RNA were detected by NanoDrop spectrophotometer and gel electrophoresis. The cDNA synthesis for liver and spleen (*n* = 5) of each group were performed by using TAKARA Bio Inc., Japan, as per manufacturer’s instruction and was stored at −80 °C for subsequent qRT-PCR.

### 2.12. Measurement of Gene Expression via qRT-PCR 

Then, qRT-PCR was performed to determine the expression of seven target genes of insulin-like growth factor-1 (*IGF-1*), growth hormone receptor (*GHR*), fibroblast growth factor 1 (*FGF1*), transforming growth factor beta-1 (*TGFB1*), T-cell immune regulator 1 (*TCIRG1*), interleukin 10 (*IL10*), and adenosine deaminase (*ADA*), in both liver and spleen tissues, using the ABI 7500 Realtime Detection System (Applied Biosystems, Foster City, CA, USA) and qRT-PCR reagents (TransGen Biotech, Beijing, China). Each 20 μL PCR reaction system contained 10 μL of 2 × TransStart Top/Tip Green Qpce, 0.4 μL (10 pM) of each primer, 0.4 μL of Passive Reference Dye (50×), 0.8 μL of cDNA (100 ng), and 8 μL of ddH_2_O. After an initial denaturing for 30 s at 95 °C, there were 40 cycles of amplification (95 °C for 15 s, 57 °C for 30 s, and 72 °C for 85 s), followed by thermal denaturing, to generate melting curves. The primers’ information for targeting genes are shown in Table 2. 

To normalize target genes, each sample for qRT-PCR was conducted in triplicate, and the experimental genes levels were quantified relative to the geometric mean of both beta-actin (*β-actin*) and glyceraldehyde 3-phosphate dehydrogenase (*GAPDH*) as two of endogenous control. Serial dilutions of PCR templates (cDNAs) were used to calculate amplification efficiency (E) for each gene (the slope values of the curves were converted to the E values, using the qPCR Primer Efficiency Calculator available at https://toptipbio.com/primer-efficiency-calculator/). The efficiency score of the primers is presented in Table 2. 

### 2.13. Statistical Analysis

All data were expressed as mean with SEM and were subjected to analysis of variance (ANOVA) in a one-way analysis of variance, using LM procedure of R software version 3.2.2, R Core [27]. The individual animal was considered as the experimental unit and included one fixed effect of source type of trace minerals in the statistical model. Duncan multiple-range tests were used to define the differences among treatments. The analyses of the relative gene expression quantification by qRT-PCR were performed by using the 2^−ΔΔCT^ method [28,29]. Comparisons between qPCR datasets were calculated by using ANOVA. All differences were considered significantly different at *p* < 0.05 and were indicated as trends when *p* < 0.10. Means comparisons were performed by using Duncan’s multiple range test.

## 3. Results

### 3.1. BW, ADG, and Carcass Traits 

As shown in Table 3, the results revealed that injecting 100 mg of Cu/ZnSO_4_, Zn/Cu-Mnt, and Zn/Cu–Thz increased (*p* < 0.05) the BW8, BW12, ADG 4-8, ADG 8-12, and ADG 4-12 compared to the controls; as an important finding here, the results of our organic complex “Zn/Cu-Thz” were better (*p* < 0.05) than those obtained with Zn/Cu-Mnt. The injection of 60 mg of Zn + 40 mg of Cu in the form of Cu/ZnSO_4_, Zn/Cu-Mnt, and Zn/Cu-Thz showed significant effects, but not consistent, on the relative weights of the carcass cuts and internal organs (*p* < 0.05) (Table 4). The group injected with Zn/Cu–Thz showed the highest (*p* < 0.05) final BW at 12 weeks and highest carcass relative weight (*p* < 0.05). Zn/Cu-Mnt and Zn/Cu-Thz treatments displayed a higher spleen weight (%) than the controls, and the Zn/CuSO_4_ treatment showed the highest (*p* < 0.05) abdominal fat (%). The liver weight in all treatments was higher than in the controls (*p* < 0.05).

### 3.2. Physical Characteristics and Microbial Abundance of MLD Muscle 

The influences of parenteral supplementation of Zn/CuSO_4_, Zn/Cu–Mnt, and Zn/Cu–Thz compared to the control group in physical characteristics of MLD and microbial abundance of the *Biceps femoris* (BF)/semimembranosus muscles are shown in Table 5. Generally, significant differences were observed between groups for six out of nine meat-quality attributes. The cooking loss of Zn/CuSO_4_ animals was higher (*p* < 0.05) than the controls and Zn/Cu–Mnt treatment. The shear force results indicated that a higher force was needed to shear the cooked meat of the Zn/Cu–Mnt treatment versus those of the controls, Zn/CuSO_4_, and Zn/Cu–Thz groups (*p* < 0.05), i.e., the treatments here did not result in any improvement in this variable. Further, significant differences were observed between raw MLD of separate groups for lightness (L*) and yellowness (b*), as well as color saturation (h˚). The results here are equivalent to most meat-quality traits (pHU, WHC, drip loss ratio after 24 h, a *, c, APC, and staphylococcus) among distinct groups (*p* > 0.05), except that the drip loss 24-hour percentage in Zn/CuSO_4_ treatment tended to be higher, and those of Zn/Cu–Mnt and Zn/Cu–Thz treatments tended to be lower (*p* > 0.05) than in controls; the same trend was obtained in the drip loss measured after 48 h of slaughter.

### 3.3. Blood Biochemical Parameters

As shown in Table 6, the Zn/Cu–Thz treatment had a lower serum MDA (*p* < 0.05) and higher GSH and CAT content (*p* < 0.05) than those in the other treatments. The same treatment (Zn/Cu–Thz) displayed the same result with respect to the serum SOD activity, which was higher (*p* < 0.05) than those in the other treatments and tended to be higher (*p* > 0.05) than the controls.

### 3.4. Profile of Essential Amino Acids in the MLD Muscle

The results of muscle EAA profile as affected by injecting the growing V-line rabbits with Zn/CuSO_4_, Zn/Cu–Mnt, and Zn/Cu–Thz are shown in Table 7. Compared to the controls, the muscle content of arginine was lower in Zn/Cu–Mnt and Zn/Cu–Thz treatments, the histidine was lower in Zn/Cu–Thz treatment, and the leucine content was lower in Zn/CuSO_4_ and Zn/Cu–Thz treatments. The muscle content of the other AAs, in terms of alanine, aspartic, glycine, serine, isoleucine, lysine, threonine, phenylalanine, tyrosine, and valine, in the tested treatments did not differ (*p* > 0.05) with the controls.

### 3.5. Villus Morphology and Morphometry

**Figure 3:**
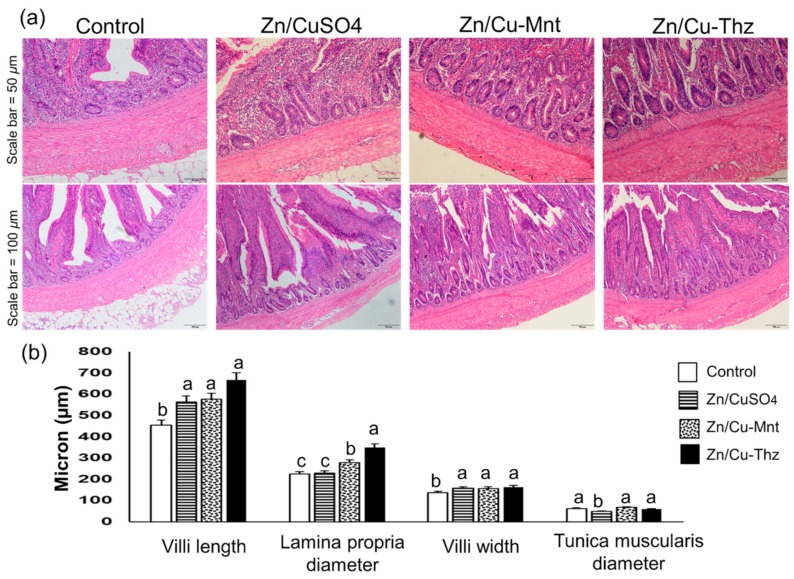
(**a**) Effect of Cu/ZnSO_4_, Cu/Zn–Mnt, and Cu/Zn–Thz on the intestinal morphology of weaned rabbits. (**b**) Morphometric analysis of villi length, lamina propria, villi width, and tunica muscularis diameter. The a, b, and c letters indicate significant differences between means at *p* < 0.05. Scale bar = 50 and 100 μm; rabbit/group, *n* = 5. Zn/CuSO_4_: zinc and copper sulfate; Zn/Cu–Mnt: zinc and copper in loaded montmorillonite; Zn/Cu–Thz: zinc and copper in hydrazone complexes.

The effects of Cu/ZnSO_4_, Cu/Zn–Mnt, and Cu/Zn–Thz injection on the rabbit villi morphology and morphometry are shown in Figure 3a,b. The results indicated that the villus length (*p* < 0.01), the lamina diameter (*p* < 0.05), and the villus width (*p* < 0.05) in the treatments were higher than in the controls, whereas the diameter of *tunica muscularis* was not affected by the treatment (*p* > 0.05). 

### 3.6. Growth Factors and Cytokine Genes Expression

**Figure 4:**
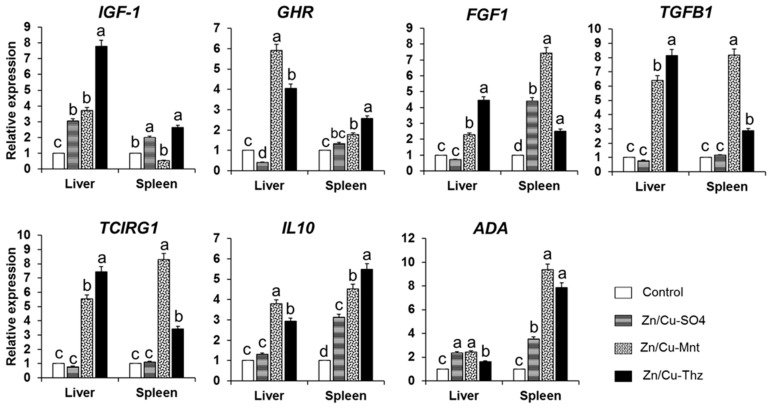
The qRT-PCR validation of mRNA expression for *IGF-1*, *GHR, FGF1, TGFB1, TCIRG1, IL10*, and *ADA* in liver and spleen tissue among groups of control, Zn/CuSO_4_, Zn/Cu–Mnt, and Zn/Cu–Thz. *IGF-1*: insulin like growth factor 1, *GHR*: growth hormone receptor, *FGF1*: fibroblast growth factor 1, *TGFB1*: transforming growth factor beta-1, *TCIRG1*: T-cell immune regulator 1, *IL10*: interleukin 10, *ADA*: adenosine deaminase. cDNA samples, liver/group (*n* = 5) and spleen/group (*n* = 5).

As shown in Figure 4, the relative mRNA expression in the tissues of liver and spleen in the Zn/Cu–Mnt and Zn/Cu–Thz treatments was significantly high compared with those of Cu/ZnSO_4_ and control. The highest liver mRNA expressions for *IGF-1*, *FGF1*, *TGFB1*, and *TCIRG1* were observed in the Zn/Cu–Thz group, whereas the highest spleen mRNA expression for *IGF-1*, *FGF1*, *TCIRG1*, *TGFB1*, and *ADA* were obtained in the Zn/Cu–Mnt treatment.

## 4. Discussion

Zn and Cu are very important for biosynthesis of more than 200 metalloenzymes and co-factors in many enzymes, regulating the physiological functions maintaining healthy and productive eukaryotic cells [30,31,32]. The use of inorganic sources of trace minerals in animal feed, currently, is cause for environmental concern, and these inorganic sources of trace minerals have poor availability in the intestinal tract due to the interaction with other minerals [33,34]. In the present study, in contrast, the supplementation with an organic source of Zn/Cu in the form of triazine hydrazone displayed a higher final BW and ADG than those of the inorganic forms (sulfate and montmorillonite). A previous study, compared between the efficiency of organic and inorganic resources of trace minerals, indicated that the higher mineral bioavailability was observed with the organic source compared to the inorganic ones [1,2,3,33]. Likewise, there was considerable digestion and absorption of minerals with a nutritional organic source of trace minerals (zinc, copper, manganese, iron, calcium, and phosphorus) compared to an inorganic source in pigs [34]. To our knowledge, there have been several reports that showed varied responses of animals supplemented with Zn and Cu from different sources [1,2,3,35,36], but the triazine hydrazone complexes had not been investigated yet. Our findings, therefore, could contribute a new potential organic complex of trace minerals that would facilitate the research of the growth-promoting products and develop some novel pharmacological drugs.

The increase in growth rate obtained here with the new complexes goes in harmony with the results of villus morphology and morphometry, which were better than the controls. Supporting results were reported, where using different organic sources of trace minerals, including Zn and Cu, improved the villus length, villus width, and crypt depth in broiler chickens [37,38] and weaned rabbits [39] than the inorganic forms. Zinc is known to have an important function in cell proliferation and differentiation, especially in the regulation of DNA synthesis and mitosis [40]. In rats, Southon et al. [41] found that the Zn deficiency caused a significant reduction in jejunal villus height, whereas Zn supplementation returned the normal morphology within a short period. These results imply that the tested complexes with improved villus morphology and growth rate had more available Zn and Cu in their intestines.

In our study, to improve the effectiveness of Zn and Cu, the Mnt was suggested as a controlled-release carrier for Cu and Zn delivery; particularly, Mnt has been utilized as an effective drug delivery carrier for sustained release of bioactive molecules, drugs, and nutrients [42,43]. Both metals here (Zn and Cu) were loaded onto Mnt through ion-exchange reactions. Moreover, the novel organic ligand (Thz) was successfully synthesized through the reaction of a hydrazino-triazine derivative and a substituted salicylaldehyde (Scheme 1). Its chemical structure was correctly confirmed from ^1^H and ^13^C NMR spectra. Two new complexes with zinc and copper (Zn–Thz and Cu–Thz) were synthesized by refluxing the ligand with equimolar amounts of metal acetate in methanol (mixed before injection) for an appropriate time (Scheme 2). 

The results here indicate that the injection of three Zn/Cu sources, Cu/ZnSO_4_, Zn/Cu–Mnt, and Zn/Cu–Thz, upregulated the expression of genes intended for growth and their enhancing effect on performance of weaning rabbits; the responses were significant, with few exceptions. The liver, in particular, is the major storage organ of Cu and Zn, and the stored Cu is largely bound to the metallothionein in most species, which have the capacity to bind both physiological (such as zinc, copper, and selenium) and xenobiotic (such as cadmium, mercury, silver, and arsenic) heavy metals through the thiol group of its cysteine residues [44,45]. The blood biochemical variables of calves were improved under Zn and Cu administration [6,46].

The obtained results in the present study showed that the relative mRNA expression for the peptide growth factors of *IGF-1*, *GHR*, *FGF1*, *TGF1*, and *TCIRG1* in the Zn/Cu–Thz treatment was higher than those in the other treatments, which implies an enhanced bioavailability of Zn and Cu. The peptide growth factors play an important role in several intracellular processes, such as cellular growth and differentiation, angiogenesis, and carcass quality [47,48,49], enhancing their expression due zinc administration [50,51,52]. Cu and Zn were higher provided from the triazine hydrazone, as liver Cu and Zn were greater with supplementation with Zn/Cu–Thz compared to those in the other treatments, but plasma Cu and Zn levels were not affected by the treatment. This observation suggests greater availability of Zn/Cu with the complex Zn/Cu–Thz, which could be attributed to the high utilization rate in enhancing the growth performance and deposition rate in the animal tissues and carcass weight obtained in this treatment.

The current study recorded pHU values of intermediate range compare to the results by [53]. Meat quality attributes, including WHC, drip loss, shear force, and carcass color, are always impacted by pH level [54]. Statistically, the pHU and WHC were not significantly influenced by combined Zn/Cu complexes, yet WHC values were still higher in the tested treatments than in the control. This was partially in accordance with the results of [55], who used different dietary Zn and Cu forms in poultry. Additionally, drip loss_24h_ and drip loss_48h_ percentages generated from supplemented rabbits were close to those in the controls and seems to be rather constant (*p* > 0.05). Supporting results were reported by Liu, et al. [56], who used Zn in broilers. Moreover, cooking-loss percentages negatively increased by Zn/CuSO_4_ than in the controls (*p* < 0.05). In contrast, the Zn/Cu–Mnt and Zn/Cu–Thz treatments here, as well as those in previous studies, which tested various complexes of Zn, positively reduced the cooking loss [57]. In terms of meat tenderness, no differences between treatments were observed, but Zn/Cu–Mnt produced higher WBSF value than in other treatments (*p* < 0.05), which mean tougher meat. In addition, Zn/Cu–Mnt lowered the meat lightness, yellowness, and color saturation of MLD than their controls (*p* < 0.05), which implies darker meat. Meanwhile, the Zn/CuSO_4_ and Zn/Cu–Thz injections did not influence any of the meat color scores, compared to the controls; and this is consistent with the results of Yang et al. [57] in broilers’ meat. In the present study, the APC and Staphylococcus count (CFU/g), similarly, did not differ (*p* ≥ 0.05) due to parenteral supplementation with Zn/CuSO_4_, Zn/Cu–Mnt, and Zn/Cu–Thz in respect to the control group (Table 5).

In the current study, injected rabbits with Zn/Cu–Thz showed lower serum MDA concentrations than those injected with Zn/CuSO_4_ and Zn/Cu–Mnt, as well as in the control group (Table 6). The serum concentration MDA is a generally indicator of the lipid peroxidation activity in cells. On the other hand, the highest activities of GSH, CAT, and SOD were observed with serum of Zn/Cu–Thz group, which generally thought to act as enzymatic free-radical scavengers in cells, dissipating of O_2_ to H_2_O_2_ and oxygen and then scavenge H_2_O_2_ from cells for removing reactive oxygen species (ROS). Antioxidant enzymes function as a defensive system against deleterious free radicals in cells, to enhance immunity, health, and growth response, consistent with the results reported by Sahin et al. [58]. Therefore, the high weight gain in the Zn/Cu–Thz group led to a decrease in some amino acid content in MLD (Table 7). In addition, alanine, arginine, glycine, serine, and histidine decreased in the Zn/Cu–Thz group compared to the control group, which may be consistent at a low concentration of dry matter in MLD with weight-gain activity [59].

## 5. Conclusions

Parenteral supplementation of growing V-line rabbits with Zn/Cu complexes, regardless of the source, did not negatively affect meat quality traits compared to the controls. Additionally, the results indicated that our novel synthesized Zn/Cu complexes loaded onto triazine hydrazone could be a suitable feed supplement to increase productive performance activities through enhancing villus morphology and expression of peptide growth factors and cytokine genes.

## Figures and Tables

**Table 1:** Composition and calculated analyses (g/kg) of the experimental diet.

Ingredients	Content
Alfalfa hay	350
Yellow corn	200
Soybean meal	96
Wheat bran	300
Corn Stover	30
Di-calcium phosphate	12.5
Sodium chloride	5
L-Lysine HCl	2.5
DL-Methionine	2
Vitamin/mineral premix ^a^	2
Total	1000.0
Calculated analysis (g/ kg on dry matter basis)	
Digestible energy (MJ/kg)	11.6
Crude protein	179
Crude fiber	125
Crude fat	32.0
Ca	10.9
Lysine	9.0
Available P	5.9
Methionine	4.2

^a^ Contained per kg of diet: 2200 IU Vit. D3; 12,000 IU Vit. A; 10 IU Vit. E; 1.0 mg Vit. B1; 2.0 mg Vit. K; 4.0 mg Vit. B2; 0.001 mg Vit. B12; 1.5 mg Vit. B6; 6.7 mg Pantothenic acid; 1.07 mg Biotin; 1.67 mg Folic acid; 10.0 mg Niacin; 400 mg Choline chloride; 80.0 mg Mn; 25.0 mg Fe; 50.0 mg Zn; 8.0 mg Cu; 2.0 mg I; 0.1 mg Se, and 133.4 mg Mg.

**Table 2:** Primers used for quantitative real-time PCR analysis of genes expressions.

Gene Name	Gene Bank Accession Number	Primer Sequence (5′–3′)	Product Length (bp)	Amplification Efficiency (E Value)
*IGF-1*	NM_001082026.1	F: AACAAGCCCACAGGATACGG	98	1.97
		R: TCCAGCCTCCTCAGATCACA		
*GHR*	NM_001082636.1	F: ACGTGTCGAGCCAAGCTTTA	91	1.91
		R: GTCTTCTGCTGTCCCAGACC		
*FGF1*	NM_001171488.1	F: GTGTTTGTTCCTGGAACGGC	98	2.00
		R: CGTTTTTCTTCAGCCCCACG		
*TGFB1*	XM_008249704.2	F: TGTCCACCTGCAAGACCATC	86	2.02
		R: CCGCAGTTTGGACAGGATCT		
*TCIRG1*	AF393372.1	F: TGTCCACCTGCAAGACCATC	86	1.96
		R: CCGCAGTTTGGACAGGATCT		
*IL10*	XM_008268045.2	F: AGCTCTGCTATGTTGCCTGG	94	1.96
		R: GCCTGGAAAGTGAATGCAGC		
*ADA*	XM_002721061.3	F: TCAAGAAGGACCAGGCGAAC	108	2.01
		R: CAGTAAAGCCCATGTCCCGT		
*GAPDH*	NM_001082253.1	F: GTCAAGGCTGAGAACGGGAA	95	2.00
		R: CCAGCATCACCCCACTTGAT		
*β-actin*	NM_001101683.1	F: CGCAAGTACTCGGTGTGGAT	94	2.02
		R: CCGACTCGTCATACTCCTGC		

*IGF-1*: insulin-like growth factor 1; *GHR*: growth hormone receptor; *FGF1*: fibroblast growth factor 1; *TGFB1*: transforming growth factor beta-1; *TCIRG1*: T-cell immune regulator 1; *IL10*: interleukin 10; *ADA*: adenosine deaminase; *GAPDH*: glyceraldehyde-3-phosphate dehydrogenase; *β-actin*: beta-actin.

**Table 3:** Nutritional impacts of dietary Zn/Cu loaded in montmorillonite (Mnt) and triazine hydrazone (Thz) complexes on growth performance of weaned V-line rabbits aged 4–12 weeks.

Growth Parameters	Type of Zn and Cu Supplementation ^b^				SEM	*p*-Value
	Control	Zn/CuSO_4_	Zn/Cu–Mnt	Zn/Cu–Thz		
BW4 (g)	498.86	517.93	497.46	506.01	7.95	0.257
BW8 (g)	834.66 ^c^	908.11 ^c^	1038.05 ^b^	1112.33 ^a^	16.48	0.001
BW12 (g)	1533.66 ^c^	1626.94 ^c^	1689.33 ^b^	1862.46 ^a^	15.61	0.001
ADG8-4 (g/d)	12.31 ^c^	13.93 ^c^	19.30 ^b^	21.65 ^a^	2.56	0.001
ADG12-8 (g/d)	24.64 ^bc^	25.66 ^ab^	23.26 ^c^	26.79 ^a^	1.89	0.048
ADG12-4 (g/d)	36.95 ^c^	39.57 ^c^	42.56 ^b^	48.44 ^a^	3.62	0.058

All data are expressed as the mean with SEM in same row; ^a, b, c^ letters indicate significant differences between means (*p* < 0.05). Rabbit/group, *n* = 15; Zn/CuSO_4_: zinc and copper sulfate; Zn/Cu-Mnt: zinc and copper in loaded montmorillonite; Zn/Cu–Thz: zinc and copper in hydrazone complexes; BW4: initial body weight at four weeks; BW8: body weight at eight weeks; BW12: final body weight at 12 weeks; ADG8-4: average daily gain from 4 to 8 weeks; ADG8-12: average daily gain from 8 to 12 weeks; ADG4-12: average daily gain from 4 to 12 weeks.

**Table 4:** Nutritional impacts of dietary Zn/Cu loaded in montmorillonite (Mnt) and triazine hydrazone (Thz) complexes on the relative weights of carcass cuts and internal organs of weaned V-line rabbits aged 12 weeks.

Parameters	Type of Zn and Cu Supplementation ^b^				SEM	*p*-Value
	Control	Zn/CuSO_4_	Zn/Cu–Mnt	Zn/Cu–Thz		
Live body weight (g)	1341.62 ^c^	1436.81 ^c^	1593.60 ^b^	1836.40 ^a^	49.25	0.001
Carcass rate (%)	55.59 ^a^	50.07 ^ab^	48.16 ^b^	55.05 ^a^	1.87	0.021
Adipose fat rate (%)	1.64 ^ab^	1.81 ^a^	1.46 ^b^	1.53 ^b^	1.05	0.029
Hind legs rate (%)	9.61 ^b^	10.34 ^b^	12.92 ^a^	10.19 ^b^	1.74	0.028
Saddle rate (%)	8.78 ^c^	10.44 ^b^	12.02 ^a^	9.89 ^bc^	1.44	0.001
Fore legs rate (%)	6.37	6.46	6.89	6.61	0.33	0.712
Thoracical neck rate (%)	6.50 ^b^	6.65 ^b^	8.04 ^a^	7.12 ^ab^	0.43	0.079
Liver index (%)	2.26 ^b^	3.75 ^a^	3.29 ^a^	3.19 ^a^	0.37	0.048
Kidney index (%)	0.99	1.06	0.96	0.90	0.04	0.127
Spleen index (%)	0.08 ^c^	0.12 ^bc^	0.18 ^a^	0.13 ^b^	0.01	0.001
Lung index (%)	1.88 ^a^	1.51 ^bc^	1.49 ^b^	1.43 ^b^	0.11	0.054

All data are expressed as the mean with SEM in same row; ^a, b, c^ letters indicate significant differences between means (*p* < 0.05). Rabbit sample/group, *n* = 5. The parameters rate and organ index were calculated as follows: parameters rate or organ index = (organ weight/living weight) × 100%. Zn/CuSO_4_: zinc and copper sulfate; Zn/Cu-Mnt: zinc and copper in loaded montmorillonite; Zn/Cu-Thz: zinc and copper in hydrazone complexes.

**Table 5:** Nutritional impacts of zinc and copper in loaded montmorillonite and their triazine hydrazone complexes on the physical muscle quality of weaned V-line rabbits aged 12 weeks.

Meat Quality	Type of Zn and Cu Supplementation ^b^				SEM	*p*-Value
	Control	Zn/CuSO_4_	Zn/Cu–Mnt	Zn/Cu–Thz		
pH grade	5.79	5.80	5.72	5.75	0.027	0.777
WHC	88.06	88.50	90.16	88.59	1.173	0.960
Drip loss (24 h)	1.99	2.20	1.63	1.60	0.105	0.065
Drip loss (48 h)	2.50 ^ab^	3.07 ^a^	1.91 ^b^	2.19 ^b^	0.175	0.029
Cooking loss	11.81 ^b^	18.05 ^a^	12.01 ^b^	16.01 ^ab^	1.093	0.041
WBSF	3.85 ^b^	3.33 ^b^	4.79 ^a^	3.32 ^b^	0.160	0.003
Lightness (L*)	55.21 ^ab^	56.73 ^a^	53.21 ^c^	53.76 ^bc^	0.449	0.007
Redness (a*)	11.48	10.56	10.95	10.55	0.230	0.474
Yellowness (b*)	5.0975 ^a^	4.8 ^a^	3.7125 ^b^	5.18 ^a^	0.189	0.005
Color Chroma (c)	12.58	11.60	11.56	11.76	0.232	0.400
Hue angle (h˚)	24.01 ^a^	24.46 ^a^	18.80 ^b^	26.19 ^a^	0.899	0.006
APC (CFU/g)	3.58	4.00	4.28	3.88	0.137	0.409
Staphylococcus CFU/g	3.31	3.95	3.60	3.27	0.123	0.137

All data are expressed as the mean with SEM in same row; ^a, b, c^ letters indicate significant differences between means (*p* < 0.05). Rabbit sample/group, *n* = 5. Zn/CuSO_4_: zinc and copper sulfate; Zn/Cu–Mnt: zinc and copper in loaded montmorillonite; Zn/Cu–Thz: zinc and copper in hydrazone complexes; WHC: water holding capacity; WBSF: Warner–Bratzler shear force; APC: total aerobic plate count.

**Table 6:** Nutritional impacts of injecting zinc and copper loaded in montmorillonite and their triazine hydrazone complexes on the variables of serum antioxidant in the weaned V-line rabbits aged 12 weeks.

Antioxidant Variables ^a^	Type of Zn and Cu Supplementation ^b^				SEM	*p*-Value
	Control	Zn/CuSO_4_	Zn/Cu–Mnt	Zn/Cu–Thz		
MDA (nmol/mL)	19.82 ^a^	18.07 ^a^	20.18 ^a^	12.67 ^b^	1.51	0.013
GSH (μmol/mL)	4.75 ^b^	4.70 ^b^	5.89 ^b^	7.14 ^a^	0.80	0.045
CAT (μmol/mL)	18.18 ^b^	12.86 ^c^	17.44 ^bc^	23.52 ^a^	1.63	0.002
GSSG (μmol/mL)	0.50	0.45	0.46	0.44	0.03	0.642
SOD (Ul/µmL)	54.18 ^ab^	48.32 ^b^	51.64 ^b^	63.64 ^a^	3.51	0.039
AMP (μg/mL)	6.62	7.08	7.67	5.58	0.71	0.667
8-OHdG (pgl/mL)	73.72	72.54	87.56	77.65	4.96	0.172

All data are expressed as the mean with SEM in same row; ^a, b, c^ letters indicate significant differences between means (*p* < 0.05). Rabbit sample/group *n* = 5. Zn/CuSO_4_: zinc and copper sulfate; Zn/Cu–Mnt: zinc and copper in loaded montmorillonite; Zn/Cu–Thz: zinc and copper in hydrazone complexes; MDA: Malondialdehyde; ADP: Adenosine monophosphate; GSH: reduced glutathione; GSSG: Oxidized glutathione; CAT: catalase; SOD: superoxide dismutase; 8-OHdG: 8-hydroxy-2’ –deoxyguanosine; AMP: Adenosine monophosphate.

**Table 7:** Nutritional impacts of zinc and copper in loaded montmorillonite and their triazine hydrazone complexes on the essential amino acid profile in MLD basis on dry matter of weaned V-line rabbit aged 12 weeks.

Amino Acid ^a^	Type of Zn and Cu Supplementation ^b^				SEM	*p*-Value
	Control	Zn/CuSO_4_	Zn/Cu–Mnt	Zn/Cu–Thz		
Alanine	25.60 ^ab^	27.15 ^a^	22.46 ^b^	25.12 ^ab^	2.04	0.041
Arginine	19.45 ^a^	17.68 ^ab^	15.62 ^bc^	13.74 ^c^	0.77	0.001
Aspartic	7.79	7.91	7.04	6.96	0.45	0.343
Glycine	83.73	82.18	71.12	72.43	4.34	0.055
Serin	13.67 ^ab^	14.52 ^a^	11.76 ^b^	11.52 ^b^	0.79	0.048
Histidine	7.30 ^a^	6.76 ^a^	6.55 ^a^	5.24 ^b^	0.32	0.003
Isoleucine	7.96	8.88	8.31	8.06	0.33	0.247
Leucine	17.03 ^a^	14.07 ^b^	15.83 ^ab^	14.15 ^b^	1.81	0.050
Lysine	33.33	29.69	31.76	33.47	1.45	0.260
Threonine	3.04	3.09	2.27	2.95	0.14	0.406
Phenylalanine	14.78	16.56	14.81	14.92	0.69	0.243
Tyrosine	14.87	14.24	13.50	14.28	0.63	0.516
Valine	15.80	16.54	16.35	16.18	0.82	0.972

^a^ Muscle amino acids content per mmol/g tissue; ^b^ all data are expressed as the mean with SEM in same row; ^a, b, c^ letters indicate significant differences between means (*p* < 0.05). Rabbit sample/group, *n* = 5. Zn/CuSO_4_: zinc and copper sulfate; Zn/Cu–Mnt: zinc and copper in loaded montmorillonite; Zn/Cu–Thz: zinc and copper in hydrazone complexes.
